# Improvement in the quality of hematopoietic prostaglandin D synthase crystals in a microgravity environment

**DOI:** 10.1107/S0909049510037076

**Published:** 2010-11-05

**Authors:** Hiroaki Tanaka, Toshiharu Tsurumura, Kosuke Aritake, Naoki Furubayashi, Sachiko Takahashi, Mari Yamanaka, Erika Hirota, Satoshi Sano, Masaru Sato, Tomoyuki Kobayashi, Tetsuo Tanaka, Koji Inaka, Yoshihiro Urade

**Affiliations:** a Confocal Science Inc., Japan; b Osaka Bioscience Institute, Japan; c Maruwa Foods and Biosciences Inc., Japan; d Japan Aerospace Exploration Agency, Japan

**Keywords:** prostaglandin D synthase, hematopoietic, H-PGDS, crystal, microgravity, space experiment, anti-inflammation, anti-allergy, JAXA, counter-diffusion

## Abstract

Human hematopoietic prostaglandin synthase, one of the better therapeutic target enzymes for allergy and inflammation, was crystallized with 22 inhibitors and in three inhibitor-free conditions in microgravity. Most of the space-grown crystals showed better X-ray diffraction patterns than the terrestrially grown ones, indicating the advantage of a microgravity environment on protein crystallization, especially in the case of this protein.

## Abstract 2

Crystals of hematopoietic prostaglandin D synthase grown in microgravity show improved quality.

## 1. Introduction

Hematopoietic prostaglandin (PG) D synthase (H-PGDS; EC 5.3.99.2) is a clinically important protein that is widely distributed in hematopoietic systems, and produces PGD_2_ from cyclooxygenase-derived PGH_2_ (Kanaoka & Urade, 2003 ▶). PGD_2_ mediates allergic and inflammatory reactions through two distinct types of receptors: the D-type prostanoid receptor (DP) and the chemoattractant receptor-homologous molecule expressed on Th2 cells (CRTH2). Although indomethacin and aspirin are known to suppress allergic and inflammatory reactions, they inhibit the production of all PGs including cytoprotective and anti-inflammatory PGs (Takeuchi *et al.*, 2001 ▶; Halter *et al.*, 2001 ▶). Therefore, selective inhibitors of H-PGDS are considered to be more useful candidates for anti-allergic and anti-inflammatory drugs as they suppress only the signals mediated by DP and CRTH2.

H-PGDS has been crystallized in a microgravity environment in Japan Aerospace Exploration Agency (JAXA)’s space experiments several times since 1997 as summarized in Table 1 ▶. Although it was not easy to obtain good crystals in earlier space experiments, high-quality crystals were obtained after JAXA improved crystallization technology and increased the accessibility of space experiments during a course of high-quality protein crystallization experiments on the International Space Station (ISS) starting in 2003 (Sato *et al.*, 2006 ▶). Recently, Takahashi *et al.* (2010 ▶) reported that crystals of H-PGDS in complexes with novel inhibitors, which were grown in microgravity in 2007, diffracted up to a 1.1 Å resolution at 100 K using SPring-8 synchrotron radiation, one of the highest resolutions obtained to date for this protein. These novel inhibitors were derivatives of 4-benzhydryloxy-1-[3-(1*H*-tetrazol-5-yl)-propyl]-piperidine (HQL-79) which is an orally active anti-allergic drug and specifically inhibits the biological activity of H-PGDS (Aritake *et al.*, 2006 ▶). Moreover, the novel inhibitors had more than 100 times lower inhibitory concentrations (IC_50_) than HQL-79, so they are expected to be candidates for novel drug design.

In this report we show more crystallization results of H-PGDS in four recent space experiments performed from 2007 to 2009, and indicate that microgravity positively affects the solution environment improving the quality of the crystals of this protein.

## 2. Materials and methods

### 2.1. Microgravity experiments

H-PGDS was crystallized at 293 K inside the Russian TBU incubator on board the Russian Service Module on the ISS for 12, 11 and 10 weeks in JAXA-NGCF#1, #2 and #3, respectively, and inside the Protein Crystallization Research Facility on board the Japanese Experiment Module ‘Kibo’ on the ISS for 12 weeks in JAXA PCG#1 as shown in Table 1 ▶.

### 2.2. Protein expression, purification and crystallization

Human H-PGDS was expressed and purified as previously described (Aritake *et al.*, 2006 ▶; Takahashi *et al.*, 2010 ▶). The crystallization conditions were as follows: a protein solution contained 4.0 mg ml^−1^ protein with or without 0.5 m*M* inhibitor in 150 m*M* sodium chloride, 15% PEG 6000, 5 m*M* dithiothreitol, 5 m*M* glutathione, 1% dioxane, 0.5 m*M* magnesium chloride and 20 m*M* Tris-HCl pH 8.0, and a precipitant solution contained 30% PEG 6000, 10 m*M* dithiothreitol, 10 m*M* glutathione, 1% dioxane and 1 m*M* magnesium chloride in 50 m*M* Tris-HCl pH 8.4. For other inhibitor-free conditions the divalent metal ion, magnesium chloride, was replaced by calcium chloride or a chelating agent EDTA.

For crystallization, JAXA adopted a gel-tube method for microgravity experiments, which modified the original counter-diffusion method introduced by García-Ruiz & Moreno (1994 ▶) (García-Ruiz, 2003 ▶; Ng *et al.*, 2003 ▶; Tanaka *et al.*, 2004*a* ▶; Gonzalez-Ramirez *et al.*, 2008 ▶; Otálora *et al.*, 2009 ▶). Assembly of a crystallization device and a micro-seeding technique were described previously (Takahashi *et al.*, 2010 ▶). Briefly, 8 µl of the protein solution was put in each glass capillary (47 mm length, 0.5 mm diameter) to a length of 40 mm. A 6 mm piece of plastic tubing was attached to the end of the capillary, which contained 1% polymerized agarose gel pre-soaked in 15% PEG 6000, 10 m*M* dithiothreitol, 10 m*M* glutathione, 2% dioxane, 1 m*M* magnesium chloride (calcium chloride or EDTA) and 50 m*M* Tris-HCl pH 8.4. The top of the capillary was sealed with clay and epoxy adhesive. The agarose end of the capillary was placed in a tube which contained the precipitant solution. Each protein sample was loaded into three capillaries. A total of 22 kinds of inhibitors of H-PGDS were co-crystallized. The formulae of the inhibitors are proprietary information. Crystallization conditions were fixed to start crystallizing after the samples arrived in the microgravity environment. The same crystallization condition was applied to the terrestrial experiment as the control.

### 2.3. Data collection and analysis

Diffraction data were collected from a single crystal at 100 K using an X-ray wavelength of 0.85 Å on the BL41XU beamline at SPring-8, Harima, Japan, with an ADSC315 detector system, or using an X-ray wavelength of 1.0 Å on the X06SA beamline at the Paul Scherrer Institute (PSI), Villigen-PSI, Switzerland, with a MAR225 detector system. The methods of crystal extraction from capillaries and harvesting crystals were previously described (Tanaka *et al.*, 2004*a* ▶, 2007 ▶). Prior to data collection, a crystal was scooped with a nylon loop, briefly soaked in an artificial mother liquor supplemented with 15% glycerol as a cryoprotectant, and plunged into a nitrogen-gas stream at 100 K. The diffraction images collected at SPring-8 were integrated and scaled using the programs *DENZO* and *SCALEPACK* from *HKL2000* (Otwinowski & Minor, 1997 ▶), and the images collected at PSI using *XDS* (Kabsch, 1993 ▶). X-ray diffraction data for each sample were obtained from two to three crystals with approximate dimensions of 0.03 × 0.1 × 0.05 mm. A summary of the best data is listed in Table 2 ▶. Data sets were collected up to the resolution range *I*/σ(*I*) > 2 and *R*_merge_ < 50%.

## 3. Results and discussion

### 3.1. Crystallization and X-ray diffraction analysis

The sizes of the crystals grown in space were about the same or sometimes significantly larger than those grown on the ground and several times larger than those grown with the vapour-diffusion method. All the crystals chosen for diffraction data collection were grown in the upper part of the capillaries where crystals of better quality tend to grow (Lopez-Jaramillo *et al.*, 2003 ▶), and those were of good quality as judged by a visual inspection.

As shown in Table 2 ▶, H-PGDS was crystallized under 25 different conditions in total, both in space and on the ground: 22 with an inhibitor and three without an inhibitor but with a different divalent metal ion (Mg^2+^: sample ID S/G6; Ca^2+^: sample ID S/G7) or a chelating agent (EDTA: sample ID S/G8).

Comparisons of the quality of space-grown and ground-grown crystals are invalid if X-ray diffraction experiments are not performed or if the same beamline is not used for both space-grown and ground-grown crystals of the same sample. Therefore, we must exclude S/G7, S/G12, S/G13 and S/G15–19 because X-ray diffraction experiments were, unfortunately, not performed owing to the unavailability of beam time, and S/G2 because X-ray diffraction data were collected at SLS beamline X06SA for only the space-grown crystals for this condition.

In the case of S/G20–22, X-ray diffraction experiments were not performed both for space-grown and ground-grown crystals owing to the poor shape of the crystals. In the case of S/G11, no crystallization was observed both for space-grown and ground-grown crystals.

Therefore, there are 12 samples that were valid for comparison, as shown in bold face in Table 2 ▶. Among these samples, S/G24 and S/G25 showed improvement of crystallizability in space-grown crystals, which was indicated by the size and the shape of the crystals, respectively. The microgravity environment improved the maximum resolution and mosaicity in S/G1, S/G3, S/G4, S/G6, S/G8–10 and S/G14. In the case of S/G5, the microgravity environment improved the maximum resolution but not the mosaicity. For S/G23, the microgravity environment did not improve the maximum resolution but did improve mosaicity.

### 3.2. Microgravity effects

The microgravity environment definitely had a positive effect on the growth of high-quality H-PGDS crystals as shown by the better results of space-grown crystals of 12 comparable samples. When comparing two to three crystals of each sample, a superior quality improvement of space-grown crystals was mostly observed, although there were some deviations in the X-ray diffraction data quality of crystals grown both in space and on the ground. The absence of convective fluid motion in microgravity is known to decrease protein concentration around the growing crystal, forming a protein depletion zone (PDZ) (Otálora *et al.*, 2001 ▶). The slower growth of crystals in the PDZ is thought to be preferable for a better-ordered intermolecular arrangement in a crystal, which may be one of the reasons for the improved quality of space-grown crystals. Impurity incorporation also affects the intermolecular order (Dold *et al.*, 2006 ▶). An impurity depletion zone (IDZ) can also be expected to form around the growing crystal (Chernov, 1998 ▶), which may reduce the incorporation of impurity molecules into the crystals in space (Thomas & Chernov, 2001 ▶).

Tanaka *et al.* (2004*b* ▶) previously reported that *D*/β (diffusive coefficient of a protein molecule/kinetic constant for crystal growth), which represents the ratio between ‘the molecule transportation to a surface of a crystal’ *versus* ‘its uptake into a crystal’ (Chernov, 1998 ▶), can be used as an index of both PDZ and IDZ. In short, if *D*/β is less than the size of the growing crystal it means that depletion zones would be formed around the crystal in a diffusion-controlled environment. Since the measurement of *D* and β is not easy to calculate for most of the proteins, we applied a simplified method of estimating those values of H-PGDS, using the molecular weight of the protein and the precipitant, the concentration of the crystallization solution, the time to grow to half of the final crystal size, and the concentration and the solubility of the protein (Tanaka *et al.*, 2004*b* ▶). We calculated that *D*/β of H-PGDS is 0.39 mm which is almost the comparative order to the crystal size. This may indicate that the H-PGDS crystals obtained in the space experiments were grown surrounded by a PDZ and IDZ, which may result in growing high-quality crystals in microgravity. Although the reason for the improvement of crystal quality in microgravity still remains a matter of speculation, H-PGDS is likely to be a better quality protein crystallized in microgravity. Using these high-quality X-ray diffraction data sets, a design for novel drug candidates is now underway.

## Figures and Tables

**Table 1:** H-PGDS crystallization experiments in space conducted by JAXA CVDA: commercial vapour-diffusion apparatus. HDPCG: high-density protein crystal growth. GCB: Granada crystallization box. JCB: JAXA crystallization box.

Flight #	Duration	Crystallization device	Launch/retrieval	Crystallization condition number	Details
STS-84	10 days, May 1997	CVDA	Space shuttle Atlantis	1	Space-grown and ground-grown crystals diffracted to 3.0 Å and 2.7 Å; mosaicity was 0.6 and 0.3, respectively
STS-107	16 days, January–February 2003	HDPCG	Space shuttle Columbia	1	No results owing to a tragic mission failure
NASDA-GCF#1	13 weeks, February–May 2003	GCB	Progress 10P/Soyuz 5S	1	Precipitate occurred both in space and on the ground
NASDA-GCF#2	9 weeks, August–October 2003	GCB	Progress 12P/Soyuz 6S	3	Precipitate occurred both in space and on the ground
NASDA-GCF#3	13 weeks, January–April 2004	GCB	Progress 13P/Soyuz 7S	2	Space-grown crystal diffracted to 1.3 Å for the first time (mosaicity 0.362)
JAXA-NGCF#1	12 weeks, January–April 2007	JCB	Progress 24P/Soyuz 13S	8	See Table 2 ▶
JAXA-NGCF#2	11 weeks, August–October 2007	JCB	Progress 26P/Soyuz 14S	4	See Table 2 ▶
JAXA-NGCF#3	10 weeks, February–April 2008	JCB	Progress 28P/Soyuz 15S	7	See Table 2 ▶
JAXA PCG#1	12 weeks, July–October 2009	JCB	Progress 34P/Soyuz 18S	8	See Table 2 ▶

**Table 2:** Summary of the X-ray diffraction experiments of H-PGDS crystals The best data of the X-ray diffraction experiments on two to three crystals for each complex of H-PGDS with compound are shown. Data sets were collected up to the resolution range *I*/σ(*I*) > 2 and *R*_merge_ < 50%. Data shown in bold face were used for data comparison between space-grown and ground-grown crystals (see text for details). All of the data except S2 were collected at SPring-8 BL41XU beamline.

Space experiment					Ground experiment					
Sample ID	Maximum resolution (visual observation) (Å)	Maximum resolution (data set) (Å)	Mosaicity	Space group	Sample ID	Maximum resolution (visual observation) (Å)	Maximum resolution (data set) (Å)	Mosaicity	Space group	Flight #
**S1**	**<1.10**	**1.10**	**0.623**	***P*1**	**G1**	**1.50**	**1.80**	**0.812**	***P*1**	**JAXA-NGCF#1**
S2†	1.20	1.20	0.178	*P*1	G2	2.00	–‡	1.736	*P*1	JAXA-NGCF#1
**S3**	**1.12**	**1.14**	**0.560**	***P*1**	**G3**	**2.00**	**–†**	**3.386**	***P*1**	**JAXA-NGCF#1**
**S4**	**1.90**	**1.90**	**1.334**	***P*1**	**G4**	**2.00**	**2.00**	**1.528**	***P*1**	**JAXA-NGCF#1**
**S5**	**1.30**	**1.30**	**1.707**	***P*1**	**G5**	**1.50**	**1.50**	**1.281**	***P*1**	**JAXA-NGCF#1 and #2**
**S6§**	**1.50**	**1.50**	**0.36–0.54**	***P*2_1_**	**G6§**	**1.70**	**1.75**	**1.05–2.47**	***P*2_1_**	**JAXA-NGCF#1 and #2**
S7§	1.40	1.40	1.329	*P*1	G7§		–¶			JAXA–NGCF#1
**S8§**	**1.26**	**1.30**	**1.109**	***P*1**	**G8§**	**2.50**	**2.50**	**1.305**	***P*1**	**JAXA-NGCF#1**
**S9**	**1.40**	**1.78**	**0.43–1.48**	***P*1**	**G9**	**2.00**		**–††**		**JAXA-NGCF#2**
**S10**	**1.30**	**1.50**	**0.66–1.03**	***P*1**	**G10**	**2.10**		**–††**		**JAXA-NGCF#2**
S11		–‡‡			G11		–‡‡			JAXA-NGCF#3
S12	1.80	1.90	0.60–1.12	*P*2_1_	G12		–¶			JAXA-NGCF#3
S13	1.47	1.48	0.63–1.43	*P*1	G13		–¶			JAXA-NGCF#3
**S14**	**1.40**	**1.68**	**0.61–1.01**	***P*2_1_**	**G14**	**1.66**	**1.70**	**0.61–4.48**	***P*1**	**JAXA-NGCF#3**
S15		–¶			G15		–¶			JAXA-NGCF#3
S16		–¶			G16		–¶			JAXA-NGCF#3
S17	1.80	1.80	0.35–0.93	*P*2_1_	G17		–¶			JAXA-NGCF#3
S18	1.80	–‡	–	*I*41	G18		–¶			JAXA PCG#1
S19	1.10	1.32	0.25–0.42	*P*1	G19		–¶			JAXA PCG#1
S20		–§§			G20		–§§			JAXA PCG#1
S21		–§§			G21		–§§			JAXA PCG#1
S22		–§§			G22		–§§			JAXA PCG#1
**S23**	**1.45**	**1.50**	**0.34–0.65**	***P*1**	**G23**	**1.35**	**1.50**	**0.48–1.53**	***P*1**	**JAXA PCG#1**
**S24**	**1.75**	**–††**	**0.940**	***P*1**	**G24**		**–††**			**JAXA PCG#1**
**S25**	**1.76**	**1.78**	**0.40–0.74**	***P*2_1_**	**G25**		**–§§**			**JAXA PCG#1**

† X-ray diffraction data set was collected at SLS beamline X06SA.

‡ X-ray diffraction data set was not collected because of high mosaicity.

§ Inhibitor-free condition.

¶ Beam time was not available.

†† X-ray diffraction data set was not collected because the crystals were too small.

‡‡ No crystallization was observed.

§§ X-ray diffraction experiment was not performed because the crystal was a cluster and not suitable for diffraction experiment.
